# Human driven climate change increased the likelihood of the 2023 record area burned in Canada

**DOI:** 10.1038/s41612-024-00841-9

**Published:** 2024-12-20

**Authors:** Megan C. Kirchmeier-Young, Elizaveta Malinina, Quinn E. Barber, Karen Garcia Perdomo, Salvatore R. Curasi, Yongxiao Liang, Piyush Jain, Nathan P. Gillett, Marc-André Parisien, Alex J. Cannon, Aranildo R. Lima, Vivek K. Arora, Yan Boulanger, Joe R. Melton, Laura Van Vliet, Xuebin Zhang

**Affiliations:** 1 Climate Research Division, Environment and Climate Change Canada, Toronto, ON Canada; 2 Climate Research Division, Environment and Climate Change Canada, Victoria, BC Canada; 3 Northern Forestry Centre, Canadian Forest Service, Natural Resources Canada, Edmonton, AB Canada; 4 Laurentian Forestry Centre, Canadian Forest Service, Natural Resources Canada, Ville de Québec, QC Canada; 5 Canadian Centre for Climate Services, Environment and Climate Change Canada, Victoria, BC Canada; 6 Pacific Climate Impacts Consortium, University of Victoria, Victoria, BC Canada

**Keywords:** Attribution, Climate-change impacts, Climate change

## Abstract

In 2023, wildfires burned 15 million hectares in Canada, more than doubling the previous record. These wildfires caused a record number of evacuations, unprecedented air quality impacts across Canada and the northeastern United States, and substantial strain on fire management resources. Using climate models, we show that human-induced climate change significantly increased the likelihood of area burned at least as large as in 2023 across most of Canada, with more than two-fold increases in the east and southwest. The long fire season was more than five times as likely and the large areas across Canada experiencing synchronous extreme fire weather were also much more likely due to human influence on the climate. Simulated emissions from the 2023 wildfire season were eight times their 1985-2022 mean. With continued warming, the likelihood of extreme fire seasons is projected to increase further in the future, driving additional impacts on health, society, and ecosystems.

## Introduction

The 2023 wildfire season in Canada was characterized by a record-breaking 15 million hectares (Mha) (150,000 km^2^) burned, by far the largest since the modern satellite record began in 1972, and more than double the previous record of 6.7 Mha in 1989. Unusually, wildfires occurred in almost all forested regions of the country and, at the national level, burned substantial areas almost continuously for approximately six months^1^. This unprecedented level of wildfire activity had many consequences for human and natural systems. Wildfires were responsible for several human casualties including firefighters, caused a record number of community evacuations (232,000 people)^1^, and put unprecedented strain on fire-fighting resources. Canada remained at the highest National Preparedness Level for an unprecedented 120 continuous days^2^. Short- to long-term economic consequences of these wildfires could reach several billions of dollars, including through their effects on the Canadian forest sector^3^. Millions were affected by heavy smoke and other volatile emissions that caused extreme levels of pollution in Canada and the United States. Such emissions also have consequences for the annual net carbon balance in Canada^1,4–8^.

An analysis by Jain et al.^1^ implicated early snowmelt, persistent drought in Western Canada and rapid transition to drought in Eastern Canada, as well as extremes in fire-conducive weather as some of the factors that contributed to this extraordinary fire season. Amidst the warmest year on record globally, Canada also experienced record warmth across much of the country, which contributed to 2023 being the most extreme fire weather year in Canada’s forests since at least 1940^1^. While the reported number of wildfires in 2023 was below average, the extreme area burned was due to the high number of very large wildfires, further implicating the role of extreme fire weather. Indeed, many of the individual wildfires that started in either May or June burned for more than four months under extended periods of fire-conducive weather.

Weather conditions affect wildfires primarily by modifying the moisture content of fuels that include both dead surface fuels (e.g., litter, duff) and live above-ground fuels^9^. Following ignition, strong winds and/or an unstable air column can further promote wildfire growth^10^. Daily surface weather can be used to estimate fuel moisture and potential wildfire behavior through the Canadian Fire Weather Index (FWI) System^9^, an empirical model used extensively by wildfire management operations and researchers around the globe. The FWI System has been used to characterize globally observed decreases in fuel moisture^11^ and increases in fire weather extremes^12^, with further changes projected in the future under anthropogenic climate change^13,14^.

Area burned has been increasing in Canada over the last half century^15^, with human-driven increases in temperature being the main driver^16^. Human influence on the climate has also driven the long-term increases in area burned in the neighbouring western United States^17,18^. The role of a warming and drying climate as a “top-down” driver of observed increases in area burned has been further elucidated in studies focused on western Canada^19,20^. Future projections of area burned in Canada suggest annual area burned may at least double under high levels of global temperature increase^21,22^. The direct human influence on wildfire activity is complex and operates across different time scales. While wildfire management in Canada is mostly focused on wildfire suppression, humans are responsible for about half of wildfire ignitions^23^. The increasing area burned is associated with an increase in large wildfire size and the number of lightning-caused fires, despite a decrease in human ignitions. In many areas, Indigenous cultural burning led to more frequent, less intense wildfires in the pre-settlement era, but the spatial extent of cultural burning was generally localized^24^.

Although there is a broad understanding of the relationship between climate change and wildfires, this does not directly tell us how climate change has changed the probability of a particular extreme fire season. In this study, we answer the question: what role did human-induced climate change play in driving the record-setting area burned of the 2023 wildfire season in Canada? While human influence on the climate has resulted in an increase in the likelihood of extreme fire weather conditions in Canada^25,26^, the western United States^27^, and other global regions^28–32^, here we go beyond attribution of fire weather, to consider whether human-induced climate change significantly influenced the area burned itself. This end-to-end attribution links area burned, which is directly related to impacts, to global human emissions of greenhouse gases. We supplement this end-to-end attribution with a land surface modeling analysis to link the unprecedented area burned in 2023 with the resulting emissions. Understanding if human-induced climate change altered the likelihood of Canada’s 2023 wildfire season can help inform adaptation and planning for future wildfire seasons under continually increasing global temperatures.

## Results

### Attribution of area burned

**Fig. 1:**
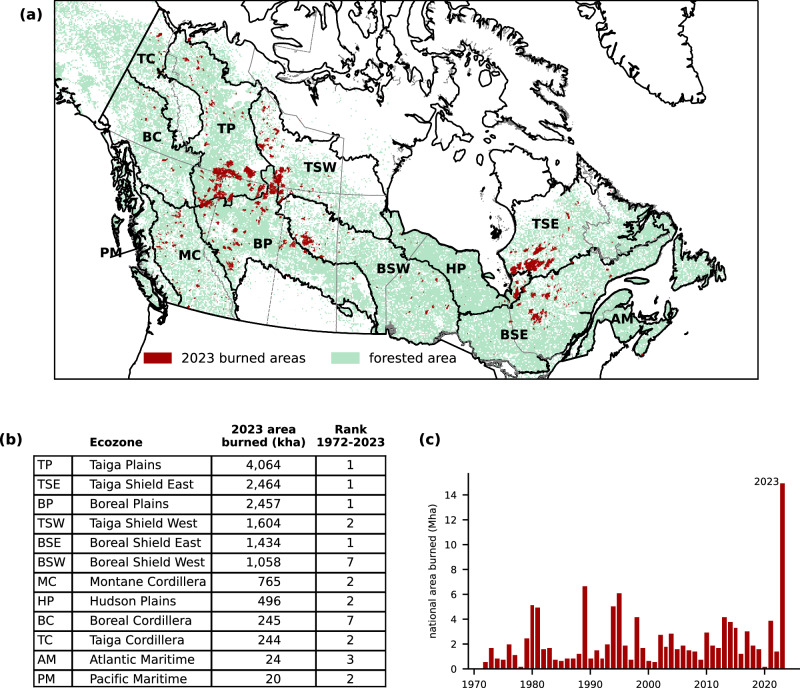
2023 area burned in Canada. **a** Map of Canada with area burned in 2023 shown in red. Forested area is shaded in light green. Ecozone boundaries are in bold and province/territory boundaries in gray. **b** Ecozone names, abbreviations, 2023 total area burned in each region, expressed in thousands of hectares (kha), and rank in the time series 1972–2023. **c** The long-term record of national area burned, expressed in millions of hectares (Mha).

The 2023 wildfire season saw large area burned across much of the forested parts of Canada, with the majority of ecozones experiencing their most or second-most extreme fire season using records beginning in 1972 (Fig. 1). In order to determine to what extent human-driven climate change influenced the likelihood of the 2023 area burned, we need large numbers of simulations of area burned with and without human influence on the climate. While some Earth System Models simulate wildfire directly^33^, these models generally underestimate burned area in Canada and other high latitude Boreal regions^33,34^. Thus, in this study we use statistical models to relate the area burned by wildfires to annual summaries of indices from the FWI System, which are based on daily temperature, precipitation, wind speed, and relative humidity (see Methods). The FWI System represents the drying of the vegetation and is the most direct link we can make between weather and wildfires. The FWI System indices have been shown to be good predictors of fire spread^35^ and area burned^36^. In this study, we develop regression models to predict area burned based on annual metrics of the FWI System indices using observations and use these to estimate area burned in simulated climates with and without human influence. Since the weather-fire relationship varies across the country^37^, much of the following analysis is performed at the ecozone level^38^, as defined in Fig. 1.

**Fig. 2:**
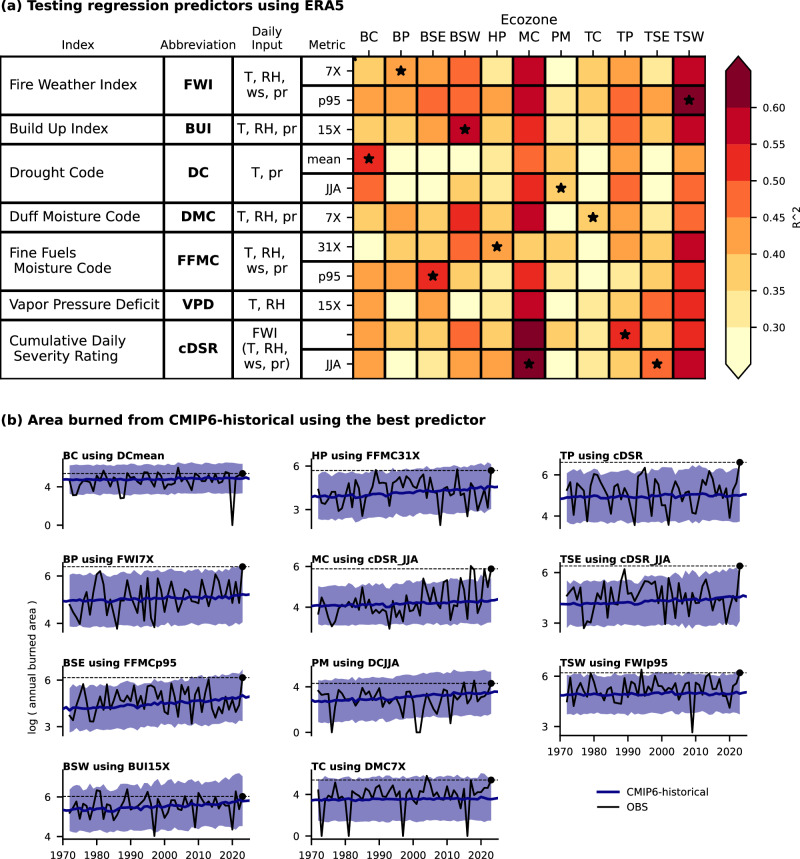
Regression models were tested and fit using ERA5 reanalysis data and observed area burned and then the best model was applied to the climate model ensembles. **a** For each ecozone (column) and potential predictor (row) from ERA5, the variance explained (R^2^) value is shown from a regression fit with the log of the observed area burned for 1972-2022. Stars indicate the predictor with the largest R^2^ for each region. For each index, additional details are listed in the table to the left, including the name and abbreviation, daily input (T: temperature, RH: relative humidity, ws: wind speed, pr: precipitation), and metric used to summarize to annual values. For the metrics, p95 refers to the 95^th^ percentile, JJA the mean (or sum in the case of cDSR) across the fire season, and 7X refers to the fire-season maximum of the seven-day running means (15X and 31X refer to the maximum of the 15-day and 31-day running means, respectively). An extended version of (**a**) is available in Supplementary Fig. 1 including more predictors. Time series (**b**) of regression-predicted area burned (log transform) for the CMIP6-historical ensemble for each ecozone. The CMIP6-historical ensemble mean is in bold blue and the shading indicates the uncertainty range, considering both model spread and regression error of prediction. The observed area burned is shown in black with a dotted line at the 2023 value.

The best predictor of the logarithm of area burned in each ecozone generally explains 40-60% of the interannual variance (Fig. 2a). The FWI System indices (Fig. 2a) are highly correlated with each other^39^, and hence there is often only a small decrease in variance explained when using the second- or third-best predictor (Fig. 2a, Supplementary Fig. 1). The regression models fitted using the best-performing predictor from the ERA5 reanalysis^40^ were then applied to four different sets of climate model simulations (Methods) of the historical climate that include human influence (in particular, increasing greenhouse gases). Three ensembles of simulations are from components of the Sixth Coupled Model Intercomparison Project (CMIP6)^41^ and the fourth ensemble is a bias-corrected large ensemble^42^. The observed time series are within the range of the predicted area burned in the model ensembles (Fig. 2b). Trends towards larger area burned over time are apparent in many ecozones in both the observations and CMIP6-historical simulations.

**Fig. 3:**
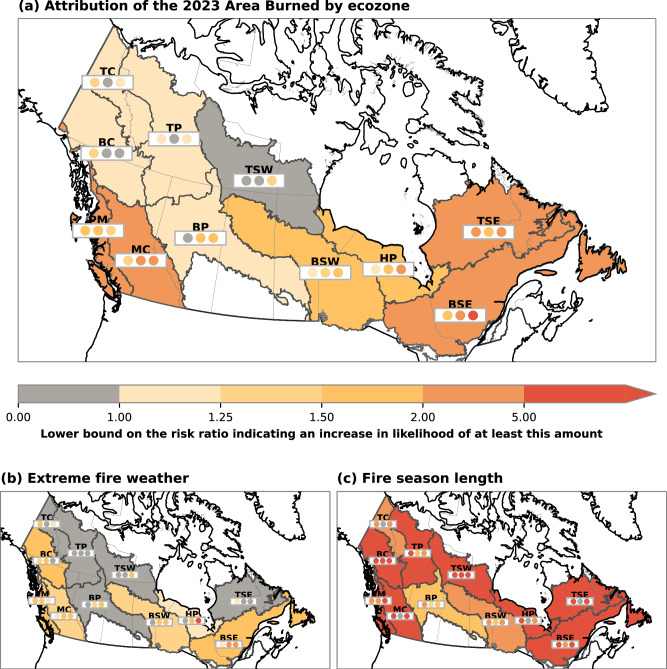
Attribution of the 2023 area burned and fire weather. Change in likelihood of an event at least as large as the 2023 (**a**) area burned, **b** maximum seven-day-mean FWI, and (**c**) fire season length by ecozone that is attributable to human influence on the climate. Each region (see Fig. 1 for the long names) is shaded according to the lower bound of the risk ratio (the ratio of the probability of the 2023 value with all forcing to the probability of the observed event in the counterfactual), based on results using CMIP6-historical. The 2023 value was extreme in many, but not all, regions. Below each region label is a set of three dots, shaded according to the same color scale, to indicate consistency of the results. These dots indicate the risk ratio using, left to right: CMIP6-DAMIP, CanLEAD-FWI, CMIP6-HighResMIP. See Supplementary Fig. 2 for the best estimate of the risk ratio.

To quantify the role of human influence on the climate in the 2023 area burned, we compare the distributions of regression-predicted area burned between the model simulations with human influence and those from a counterfactual climate with little or no human influence (Methods). A risk ratio is calculated as a ratio of the probability of an area burned at least as large as 2023 in the current climate to the probability of such an area burned in a counterfactual climate. This risk ratio represents the change in likelihood that can be attributed to human influence on the climate, with a value greater than one indicating an increase in likelihood due to climate change. Across the country, the 2023 area burned was more likely because of human influence on the climate (Fig. 3a). The 2023 area burned was made at least twice as likely by human influence on the climate in the east and southwest ecozones, including the Boreal Shield East, Taiga Shield East, Montane Cordillera, and Pacific Maritime ecozones. The attributable change in likelihood is largely consistent between climate model ensembles, increasing confidence. Several ecozones in the northwest (Taiga Cordillera, Taiga Plains, Taiga Shield West, Boreal Plains, Boreal Cordillera) demonstrated a significant attributable increase in likelihood in at least one, but not all of the four datasets, somewhat reducing confidence in the attribution in these regions. Uncertain changes in the northwest may be due to increases in summer precipitation that counteract the warming temperatures^42^ and inconsistent precipitation changes across the CMIP6 models^43^.

In addition to comparing results across different model datasets, additional sensitivity tests also contribute to confidence in the attribution of area burned. Significant increases in the likelihood of the 2023 area burned are found in most ecozones when using other indices from the FWI System as predictors (Supplementary Fig. 3). Additionally, results are largely robust to the exclusion of any one model from the multi-model CMIP6-historical ensemble (Supplementary Fig. 4). Finally, the current climate is characterized by using the global warming level in each model that corresponds to an estimate of human-induced warming based on observations (Methods). Considering uncertainty in the estimate of human-induced warming results in broadly consistent patterns of attributable increases in likelihood (Supplementary Fig. 5). In general, in areas with strong increases in the likelihood of area burned, these increases are larger for larger increases in global temperature.

### Attribution of other fire season drivers of area burned

To support the event attribution for area burned, we also applied a similar framework to the extreme fire weather and the length of the fire season (Fig. 3). A maximum seven-day FWI at least as high as 2023 was significantly more likely in the Boreal Shield East, Hudson Plains, Boreal Shield West, Montane Cordillera, Pacific Maritime, and Boreal Cordillera. Many of the regions with the largest attributable increases in the likelihood of area burned were also the regions with significant increases in the likelihood of extreme fire weather. The attributable changes in likelihood are similar for other indices in the FWI System (Supplementary Fig. 6), further increasing confidence. A fire season (see Methods) at least as long as that in 2023 was more than five times as likely because of human influence on the climate in most ecozones (Fig. 3c), with consistent results across the different datasets. Attributable increases in likelihood were also found for the 2023 start date of the fire season, although of a smaller magnitude than fire season length (Supplementary Fig. 7).

**Fig. 4:**
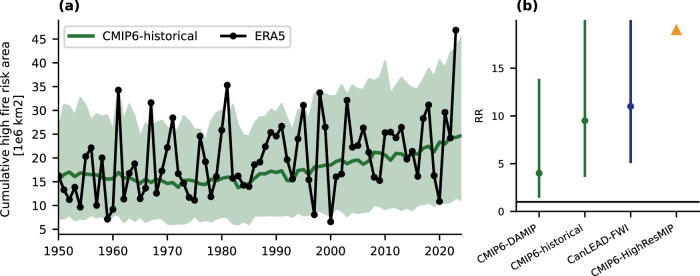
Attribution of the cumulative high fire risk area. **a** Time series of the cumulative high fire risk area calculated as the sum across the fire season of the daily forested area experiencing a Fire Weather Index value greater than its local 95th percentile based on a 1951–1980 climatology. ERA5 is shown in black and CMIP6-historical forcing in green with the ensemble means in bold and the 5th to 95th percentile range shaded. **b** Risk ratios (RR) for a cumulative area at least as large as observed in 2023 for each ensemble. The triangle for CMIP6-HighResMIP indicates an infinite RR and the upper bounds on the uncertainty range extend to infinity for CMIP6-historical and CanLEAD-FWI. An infinite RR occurs when the event in question was very rare and did not occur in the counterfactual simulations (or the resampling of them) and can be interpreted to mean that all of the likelihood of the event’s occurrence is due to human influence on the climate.

The 2023 wildfire season was extreme across most regions of the country (Fig. 1). Widespread synchronous fire-conducive weather conditions increase the national demand on wildfire management resources^44,45^. This can be considered as a spatially compound event, where extreme fire conditions occur simultaneously in multiple regions. For Canada as a whole, we calculated the cumulative high wildfire risk area by summing the area of forested grid boxes each day that exceeded their local 95th percentile of FWI (from the 1951 to 1980 base period) and then summing across the fire season^1^. The 2023 value was very large, about 177% of the 1951–1980 mean and 33% greater than the next highest year (1981). The cumulative high wildfire risk area has increased over the historical period in ERA5 and in model simulations that include human influence (Fig. 4a). This results in at least a 3.75-fold increase (best estimate of 9.5-fold) in likelihood of an area at least as high as in 2023 using the CMIP6-historical ensemble (Fig. 4b). All ensembles agree on a significant increase in likelihood, based on a lower bound of the uncertainty range on the risk ratio greater than 1. Thus, in addition to the increased likelihood of extreme fire weather in many individual ecozones, human influence on the climate has also increased the likelihood that large areas of the country will experience severe fire weather during a single fire season.

### Emissions impacts

**Fig. 5:**
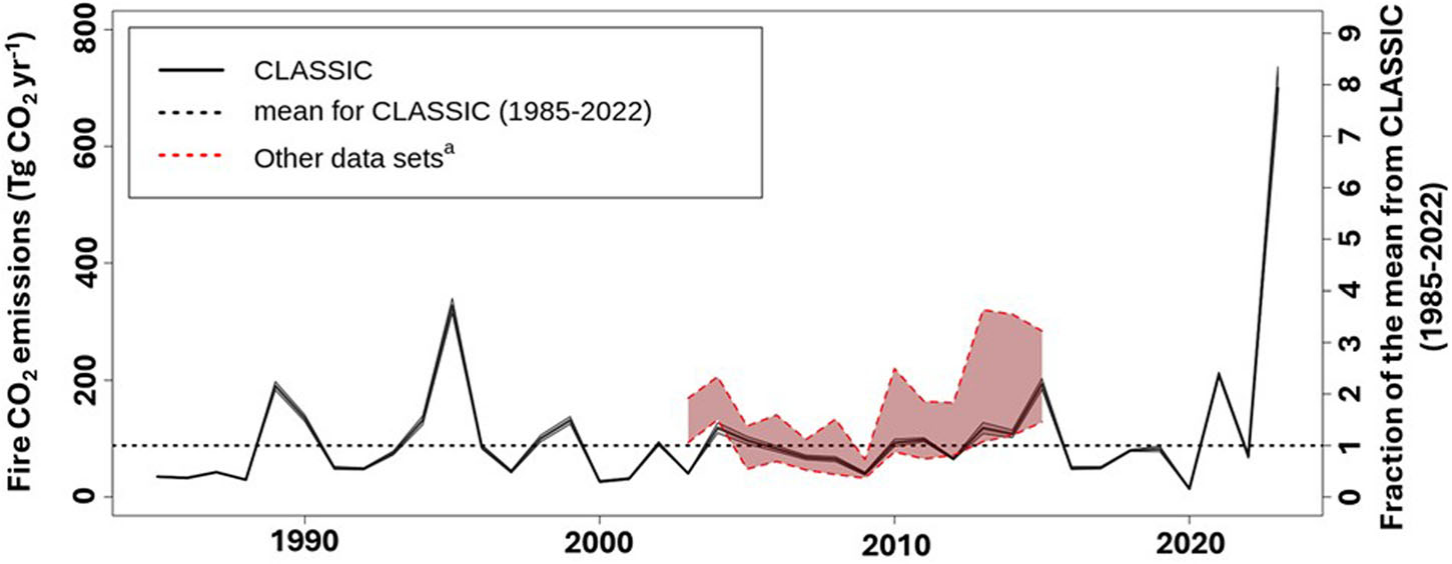
Land surface model (LSM) based fire emissions estimates. Historical fire emissions from the CLASSIC LSM from 1985 to 2023. The dashed line denotes the 1985 to 2022 LSM historical average tied to the right y-axis. The shaded region represents the standard deviation from the mean for two LSM runs using two driving meteorologies. The red-shaded region represents the annual range of five independent historical gridded estimates. ^a^Data sets include the Global Fire Emissions Database version 4.1 with small fires, the Fire Inventory from NCAR version 2.5, Fire Energetics and Emissions Research version 1.0-G1.2, the Quick Fire Emissions Dataset version 2.4 revision 1, and Carbon Tracker 2019.

The 2023 fire season released a large amount of CO_2_, which has implications for the net carbon balance in Canada and human health. We used a land surface model (LSM) to quantify wildfire CO_2_ emissions from 1985 to 2023 based on observed burned areas and simulated land surface conditions. Canada-wide wildfire CO_2_ emissions between 1985 and 2022 averaged 88 ± 62 Tg CO_2_ year^-1^. These historical estimates fall in the range of five other independent, spatially explicit estimates (Fig. 5). Annual observed burned area is highly correlated with both annual modeled emissions (Pearson *r* = 0.99) and independent historical annual emissions estimates (average Pearson *r* = 0.82 +/− 0.13, *n* = 5). The 2023 emissions were estimated at 700 Tg CO_2_, which is eight times the 1985–2022 mean (Fig. 5). This estimate is on the lower end of the range^46^ (Methods), but is consistent with observed carbon losses following fire^47^. Moreover, this emissions estimate does not consider greenhouse gas species other than CO_2_. Nonetheless, it suggests that the 2023 wildfire season in Canada was a significant source of CO_2_ emissions. With increasing warming and the accompanying increase in the likelihood of large burned areas, increases in wildfire emissions are also anticipated.

### Future changes

**Fig. 6:**
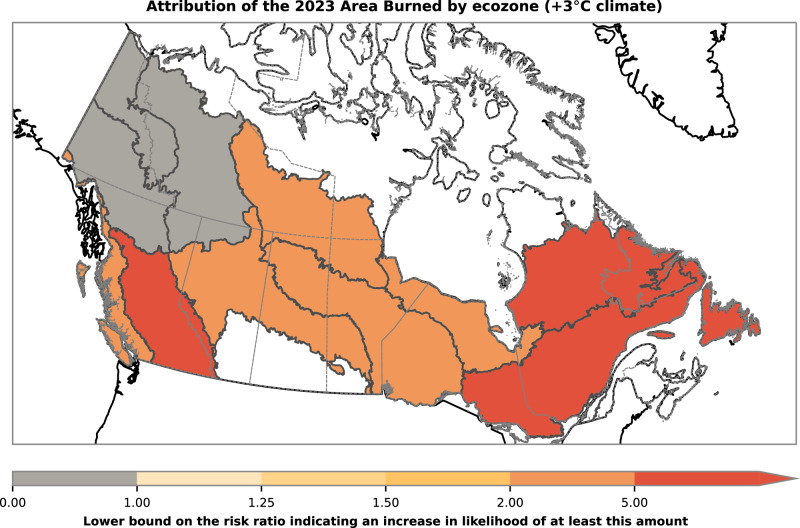
Change in likelihood of area burned for a future climate. Similar to Fig. 3a, the lower bound on the uncertainty range of the risk ratio for an area burned at least as large as that observed in 2023 is shown but for a climate with a global temperature +3 °C warmer than pre-industrial. The results shown here are using the CanLEAD-FWI dataset and compare to the center circle in Fig. 3a for the +1 °C global warming climate.

Changes in climate attributable to human influence are expected to further intensify with additional warming^48^. As such, further increases in the likelihood of long fire seasons, extreme fire weather, and large burned areas are anticipated for Canada. Fig. 6 demonstrates increases in the likelihood of an area burned at least as large as 2023 at a global warming level of +3 °C from pre-industrial, corresponding approximately to the level of global warming projected for the end-of-century under current global policies^49^. The dataset shown here is the bias-corrected regional climate model output (CanLEAD-FWI^42^) represented by the center circle in Fig. 3. Most regions are projected to experience at least a two-fold increase in the likelihood of the 2023 area burned in the future climate under high warming, with at least a fivefold increase in the regions with the strongest attributable changes in the current climate. The exception is northwest Canada, where no robust change in likelihood is projected in this dataset, which may be due to increases in summer precipitation^42^.

## Discussion

Globally, 2023 was the warmest year on record since 1850^50^, and the mean temperature in Canada during the 2023 fire season was 2.2 °C warmer than the 1991-2020 average^1^. What role did climate change play in driving the unprecedented area burned in Canada that year? Here, we have demonstrated that human influence on the climate, through anthropogenic emissions, has increased the likelihood of the large area burned observed in many regions across Canada during the record-breaking 2023 wildfire season. Our results also show that human-induced climate change increased the likelihood of the particularly extreme fire weather in 2023. A fire season as long as that in 2023 was more than five times as likely because of human influence on the climate in almost all ecozones. Longer fire seasons provide a longer window for fire-conducive weather to occur and limit the opportunities for prescribed burns for wildfire mitigation, which can lead to more large wildfires. In 2023, many very large wildfires in western Canada burned for four to five months^1^. The 2023 wildfire season impacted most forested areas of Canada and we demonstrated that the expansive areas of the country experiencing concurrent extreme fire weather were much more likely because of human influence on the climate. Widespread wildfire conditions also led to wildfire CO_2_ emissions in 2023 that were eight times the long-term modeled mean. Modeled burned area and emissions are strongly correlated at annual to decadal timescales, so the attributable impacts of anthropogenic climate change on CO_2_ emissions also follow those for area burned.

In addition to fire weather conditions, other factors also play a role in the area burned, including vegetation type, ignition sources, and wildfire management policies. These factors account for some of the unexplained variance in the regressions; however, weather represents the main control on wildfire occurrence and wildfire behavior in Canadian forests^51^. Some of these external factors correspond to non-climatic human influence (e.g., wildfire suppression or ignition, availability of fire management resources, and the associated wildfire policy)^52^, while others may be influenced by human-driven climate change (e.g., lightning frequency, changing vegetation types, increasing pests). Nevertheless, fire weather explained much of the variance of annual area burned, and statistical models based on fire weather were shown to have adequate skill.

There were regional differences in the attribution of the 2023 wildfire season. While the northern and western forested regions of Canada are prone to wildfire, with large wildfires occurring almost every year^37^, ecozones in the northwest saw smaller increases in the likelihood of area burned and extreme fire weather, with some inconsistency in attribution between different model ensembles. In addition to large interannual variability, projected increases in precipitation counteract the effects of projected warming in the CanLEAD-FWI dataset and lead to a more uncertain response of fire weather to climate change in these regions^42^. Ecozones in the east and southwest had the largest increases in the likelihood of area burned and of extreme fire weather. Regardless, the 2023 wildfire season was extreme in most ecozones, ranking first or second in the observed record beginning in 1972.

With continued global anthropogenic emissions and the resulting increase in global mean temperature, weather/climate conditions conducive to wildfires will occur more frequently and the likelihood of large burned areas will increase further in many regions. With continued warming, the strain on fire management resources required for many regions simultaneously will be an increasing concern^45^. Additionally, increasing wildfire emissions and their detrimental impacts on human health will also be an important consideration for the future.

Canada’s unprecedented wildfire season of 2023 had major impacts on communities and ecosystems at the national level^1^, and globally-significant impacts of greenhouse gas emissions and short-lived climate pollutants^50^. Our results show that not only did human-induced climate change significantly increase the likelihood of the long fire season and extreme fire weather observed in 2023, but it also significantly increased the likelihood of the large area burned in most regions of the country. Our results demonstrate how climate-change induced changes in wildfire risk are regionally differentiated across Canada, with the biggest increases in the southwest and southeast of the country, and weaker increases in the northwest due to the counteracting effects of warming and precipitation increases. Such understanding of how climate change is changing the likelihood of extreme fire seasons and large areas burned across the country can be used together with local knowledge and Indigenous Knowledge to better adapt to the climate change we are experiencing and better prepare for future fire seasons.

## Methods

### Datasets

The observed annual area burned by ecozone was calculated beginning in 1972 from the National Burned Area Composite (NBAC)^53^, high-quality spatial wildfire perimeters provided for all of Canada on an annual basis. To represent the observed fire weather conditions, we use the ERA5 reanalysis^40,54^ for 1940-2023. Several ensembles of model simulations were also used (Supplementary Table 2). These include simulations from the Coupled Model Intercomparison Project Phase 6 (CMIP6)^41^, from many MIPs under the larger CMIP6 umbrella. “CMIP6-historical” includes data from the CMIP6 historical experiment for 1950-2014, extended with the Shared Socioeconomic Pathways SSP2-4.5 scenario^55^ for 2015-2030. “CMIP6-DAMIP” includes CMIP6-historical data from a reduced set of models and the historical-natural simulations from the Detection and Attribution Model Intercomparison Project (DAMIP)^56^ for 1950-2020. “CMIP6-HighResMIP” includes data from the High Resolution Model Intercomparison Project (HighResMIP) by combining highresSST-present (1950-2014) and highresSST-future (2015-2030) experiments^57^. Unlike ScenarioMIP experiments, HighResMIP includes only one scenario, which is based on the CMIP5 RCP8.5 experiment. However, due to the similar warming behavior of the models in the period from 2015-2030 for all future scenarios, the differences between the climate projections for the CMIP6 (historical and DAMIP) and HighResMIP models are considered negligible^58^. Additionally, we also used the CanLEAD-FWI dataset^42^, which includes the FWI System indices calculated from a large initial-condition ensemble of regional climate model (CanRCM4) simulations that have been downscaled and bias corrected using a multivariate procedure^59^. CanLEAD-FWI is available for 1950-2100 and the original model forcing was a combination of historical and RCP8.5.

### Calculation of the Fire Weather Index System

The FWI System indices were calculated using the open-source Python xclim package^60^ with data preprocessed with Python-based ESMValTool software^61–64^. Conventionally, the FWI System uses local noon temperature, relative humidity, wind speed, and precipitation^9^; however, these data are not generally available for CMIP6 simulations. Van Vliet et al. ^42^ corrected daily maximum temperature and daily mean relative humidity in CanLEAD-FWI to local noon values; this correction was trained using hourly data. As hourly data was not available from most models, the CMIP6 FWI System indices were calculated using daily maximum temperature and daily minimum relative humidity as in Abatzoglou et al. ^14^ and others. The estimation of minimum relative humidity follows the procedure of CanLEAD-FWI with the assumption that this minimum occurs with the maximum temperature. For consistency, FWI System indices were calculated from ERA5 similarly to the CMIP6 models. In general, differences in the input variables result in small shifts in the absolute values of the time series for FWI System indices. However, the magnitude of the anomalies is similar between methods and so should have minimal impact on the results presented here, which employ anomalies and in-dataset comparisons. We also include the vapour pressure deficit (VPD) metric, which is not officially part of the FWI System but has been demonstrated to also well represent the extent of dry conditions relevant to wildfire potential^45^.

The fire season starts after three consecutive days with a maximum temperature exceeding 12 °C and ends after three consecutive days below 5 °C^65^. The fire season length is the number of days between the start and end of the season. An overwintering procedure^66^ is applied to determine the initial values for the longer-memory indices (DMC, DC) to start the calculations at the beginning of the fire season. This approach considers precipitation preceding the start of the fire season and the influence from interseasonal drought. We apply the same method for all of the datasets, based on the method used for the early version of CanLEAD-FWI^42^ that is used in this analysis. The DMC default start-up value of 6 is adjusted by adding 1.2 times the number of days since a rainfall event of > 1.5 mm and the DC follows McElhinny et al. ^66^ and Lawson and Armitage (2008)^67^ with the carry-over fraction and wetting efficiency fraction set to 0.75. Note that any differences between the version of CanLEAD-FWI used in this paper and the final published version due to changes in the overwintering procedure are small.

The FWI System indices were calculated at individual grid boxes on each model’s native resolution. The indices from CMIP6-HighResMIP models were interpolated to a common 0.5° grid to match the resolution of CanLEAD-FWI. With much coarser models available, the CMIP6-historical and CMIP6-DAMIP simulations were interpolated to a common 2° grid. ERA5 was interpolated to both resolutions to enable like-to-like comparisons, though the differences between the regional means calculated from the different resolutions are small. Next, area-weighted averages were calculated over the forested grid boxes in each terrestrial ecozone^38^. The forest mask is based on Hermosilla et al. ^68^ and interpolated to match the resolution of each dataset.

Individual models were evaluated by comparing climatologies to the reanalysis and to those from other models (Supplementary Fig. 8). A few models were excluded from the CMIP6-historical (KACE-1-0-G, UKESM1-0-LL, FGOALS-g3) and CMIP6-HighResMIP (NICAM16-7S, NICAM16-8S, HiRAM-SIT-HR) ensembles due to FWI System index values that were consistently much larger than other models. Across the ecozones, the reanalysis mean FWI from 1951-1980 is contained well within the spread of the means from the ensemble of model simulations.

### Fitting of regression models

Analyses were performed by region. We used the same regions as Stocks et al. ^69^, which is based on the ecozones^38^, with the Boreal Shield and Taiga Shield split into “East” and “West” subzones based on variation in the predominant wildfire regime of these large areas. An ordinary least squares regression was performed between the logarithm (base 10) of annual area burned and annual metrics of the FWI System indices from ERA5. We focus on the logarithm of area burned, because it is closer to being normally distributed than area burned itself, and this approach also avoids giving undue weight in the regression to extreme wildfire years. For each ecozone, the best predictor was identified from the full set tested based on the largest percent variance explained (R^2^). For the annual metrics, the fire season maximum of 7-, 15-, and 31-day means was calculated for all indices except the Drought Code and Daily Severity Rating, which used seasonal sums/means (Fig. 2a). The consideration of multiple averaging periods for each index allows for the depiction of regional variation in fire-fuels dynamics, such as fuel drying time, typical fire duration, and ecosystem drought resistance. Given strong correlations between potential predictors, only single-predictor models were considered. The coefficients were fitted using 1972-2022 data, intentionally leaving out the extreme year of 2023. The Atlantic Maritime ecozone was removed from the area burned analysis as no robust regression model was identified due to low area burned totals and R^2^ consistently well below 20%. To estimate area burned from the climate model ensembles, a simple bias correction was applied by subtracting the 1951-1980 mean for the FWI System predictor in each model and adding in the mean from that period in ERA5, before applying the regression coefficients. A consideration of prediction uncertainties is included in the calculation of attribution metrics, as described below. We assume that the contribution of non-climatic factors to the variance of area burned is constant in time. The R^2^ from the regressions was very similar (Pearson *r* = .988, across all predictors and regions) if the FWI System predictors and the log of area burned were first linearly detrended; following Turco et al. ^17^, we can thus conclude that non-stationary non-climatic factors did not have a notable influence on our analysis.

### Calculation of risk ratios

The risk ratio is defined as the ratio of the probability of a class of events in the factual climate to that in the counterfactual climate. The factual climate is represented by the simulated climate in each model when its global warming level matches an estimate of observed human-induced global warming (see below). CanLEAD-FWI and CMIP6-HighResMIP use the first decade of the available data (1950-1959) as a counterfactual, since anthropogenic global mean warming in that decade was only approximately 0.2 °C^70^. For comparison, CMIP6-historical risk ratios were also calculated relative to this early period with greatly reduced anthropogenic emissions and a much lower global temperature increase compared to present. For CMIP6-DAMIP, the counterfactual was calculated using the final 25 years of the natural-only forcing simulations (1996-2020), which does not include any major volcanic eruptions. In each period and for each model dataset, values from all realizations and years were pooled to determine a distribution from which the probabilities were estimated. The factual and counterfactual ensembles were constructed using the same proportional contributions of simulations from each model. We focus on the results from CMIP6-historical relative to the 1950s counterfactual, as this dataset has, by far, the largest sample size (Supplementary Table 1).

Next the probabilities were calculated for an event at least as strong as that observed in 2023 in both the factual and counterfactual periods. These probabilities were used to determine the risk ratio for each event. The threshold for estimating the probability was determined by the 2023 observed area burned or the 2023 fire weather metric anomaly from ERA5. For area burned, the probability estimation method follows Kirchmeier-Young et al. ^26^ where normal distributions were used with means equal to the regression-predicted value of area burned and standard deviation from the standard error of prediction, which accounts for regression model uncertainty. The pool of normal distributions (one from each year and realization) was combined into a mixture distribution assuming independence and equal weight to each year and realization. For the fire weather or fire season metrics, anomalies were calculated relative to the first common 30 years (1951-1980) from each model; anomalies for the natural forcing simulations were calculated relative to the same model’s historical forcing base period, to preserve the offset between the factual and counterfactual. Given the large samples when pooling realizations/years, probabilities were calculated empirically to avoid the fitting of additional parameters. The risk ratio is calculated as the ratio of the probability of the event in the factual vs. the counterfactual period. The uncertainty range on the risk ratio was estimated using the 5^th^ to 95^th^ percentile of risk ratios from a bootstrap resampling.

### Calculation of global warming levels

Climate sensitivity varies across models in CMIP6^71^. To improve the representation of the current climate, we constrain the global warming levels for individual climate models to match observed human-induced warming between the 1950s and 2023 of about 1 °C based on the methods of Forster et al. ^72^. Local climate responses to changes in external forcing are found to vary approximately linearly with global mean temperature^73^. We calculated 10-year moving anomalies relative to the counterfactual period (1950–1959) for each model. Estimated human-induced warming is an average of that calculated from three different methods^72^. We used a Gaussian distribution for human-induced warming, assuming the uncertainty is the same as in Forster et al. ^72^. We then randomly sampled (10,000 times) from the distribution at the observed human-induced warming level to determine a set of years at which each model achieves the closest warming level. Most results use the best estimate (50th percentile) from each model, but Supplementary Fig. 5 demonstrates the influence of uncertainty in the attributable warming by using the 5th and 95th percentiles. With FWI System data processed through 2030, some models do not contribute a full 10 years to the current warming level (occurs when the identified period in the model starts after 2021), resulting in risk ratios that are, if anything, slightly conservative (because warmer years are missing from some models). In any case, the number of years in the counterfactual are adjusted accordingly, so each model’s proportion of the ensemble is consistent between the current and counterfactual datasets.

### Emissions modeling

We utilized a land surface model (Canadian Land Surface Scheme Including Biogeochemical Cycles (CLASSIC))^74,75^ driven by a spatially explicit record of wildfire events to quantify wildfire emissions from 1985 to 2023. The version of CLASSIC utilized herein operates in the Canadian domain at 0.22° spatial resolution using 14 Canada-specific biogeochemical plant functional types^34^. It incorporates forced wildfire and harvest and uses the optimal model configuration with 12 dynamic tiles and a wildfire parameterization that accounts for boreal fire emissions from soil^46,76^.

Historical Canadian wildfire CO_2_ emissions estimates vary widely among methods and sources^77^. Differences in the representation of carbon emissions from burnt soil are likely the largest source of uncertainty^78^. Between 2003 and 2015 mean Canada-wide wildfire CO_2_ emissions estimated using CLASSIC are 94 ± 86 Tg CO_2_ yr^-1^. This is within the range of the means from five other spatially explicit estimates (85 - 172 Tg CO_2_ yr^-1^)^46^. Because Canada-wide emissions and burned area are strongly correlated amongst all five gridded estimates (0.72 ± 0.03), emissions intensity (carbon emissions per unit area burned) provides a more flexible uncertainty metric. The emissions intensity of 5.0 kg CO_2_ m^-2^ burned area simulated by CLASSIC again falls within the range of several gridded and field-based estimates that fall between 0.8 and 2.1 times higher on average^46^.

To evaluate the impact of forcing uncertainty on modeled wildfire emissions, we drive CLASSIC with two climate forcings and average the result. The first (GSWP3–W5E5–ERA5) forcing, is described in detail by Curasi et al. ^34^. It combines the 1901-1978 portion of the Inter-Sectoral Impact Model Intercomparison Project (ISIMIP) GSWP3–W5E5 and the 1979–2018 portion of the ERA5 time series bias corrected to match the means of the overlapping period in the GSWP3–W5E5^79,80^. The second (CRU–JRA–v2.1) forcing is derived from the blended Climatic Research Unit (CRU) and Japanese reanalysis (JRA) data^81^. The model uses land cover corresponding to the year 2010 that does not vary in time.

We compare our modeled wildfire CO_2_ emissions to estimates from several sources including the Global Fire Emissions Database version 4.1 with small wildfires, the Fire Inventory from NCAR version 2.5^82^, Fire Energetics and Emissions Research version 1.0-G1.2 (available from http://feer.gsfc.nasa.gov/data/emissions/)^83^, the Quick Fire Emissions Dataset version 2.4 revision 1 (available from https://portal.nccs.nasa.gov/datashare/iesa/aerosol/emissions/QFED/v2.4r6/)^84^ and Carbon Tracker 2019 (available from https://gml.noaa.gov/aftp/products/carbontracker/co2/)^78^.

The LSM requires a specification of the per-grid cell annual fractional area harvested or burned between 1740 and 2023, as in Curasi et al.^76^. For 2020–2023, we obtained burned area from the National Burned Area Composite time series^53^ and the National Burned Area Composite M3 interim product^1^. For the remainder of the satellite era (1985–2019), we use remotely sensed 30-m spatial resolution records of harvest and fire events derived from Landsat^85^. We spin up the model to 1700 equilibrium conditions and then carry out a 1700–2017 transient run and 2017 to 2023 run. The spin-up loops the earliest 25 years of climate data available (1901-1925) and holds atmospheric CO_2_ concentrations constant at the 1700 level. The 1700–1900 portion of the transient run loops the 1901-1925 climate but uses transient atmospheric CO_2_ concentrations. The 1900–2017 transient run uses transient atmospheric CO_2_ concentrations and evolving climate. The 2017 to 2023 portion uses looped climate starting in 2017 and looped atmospheric CO_2_ starting in 2023. During the 1740–2023 portion of the transient simulation, the fire and harvest forcings are applied.

## Supplementary information

Supplementary Material

## Data Availability

The datasets used in this manuscript are freely available. The NBAC is available from: https://cwfis.cfs.nrcan.gc.ca/datamart, ERA5 is available from: https://cds.climate.copernicus.eu/cdsapp#!/dataset/reanalysis-era5-single-levels, CMIP6 data is available from https://esgf-node.llnl.gov, CanLEAD-FWI is available at https://climatedata.ca/fire-weather/ and the underlying bias-corrected data is available at https://open.canada.ca/data/en/dataset/a97edbc1-7fda-4ebc-b135-691505d9a595.

## Code availability

Code to calculate the Fire Weather Index System indices using ESMValTool is available here: https://github.com/ESMValGroup/ESMValTool/tree/dev_cccma_extremes. Additional analysis code is available here: https://github.com/mkirchmeier/Wildfire_attribution_2023.
