# Supplementary material for: Human driven climate change increased the likelihood of the 2023 record area burned in Canada
Source: NPJ Clim Atmos Sci. 2024 Dec 20;7(1):316. doi: 10.1038/s41612-024-00841-9 (PMC11661968; doi:10.1038/s41612-024-00841-9)
Supplement: Supplementary file 1 — Supplementary Material [file 41612_2024_841_MOESM1_ESM.pdf]

SUPPLEMENTARY Tables and Figures

Human driven climate change increased the likelihood of the 2023 record area burned in Canada

Kirchmeier-Young, Megan C.[1, https://orcid.org/0000-0003-3907-8510]; Malinina, Elizaveta[2, https://orcid.org/0000-0002-4102-2877]; Barber, Quinn E.[3]; Garcia Perdomo, Karen[2,https://orcid.org/0009-0004-2333-3358,]; Curasi, Salvatore R.[2,https://orcid.org/0000-0002-4534-3344];Liang, Yongxiao[2]; Jain, Piyush[3,https://orcid.org/0000-0002-0471-4663]; Gillett, Nathan P.[2]; Parisien, Marc-André[3,https://orcid.org/0000-0002-8158-7434]; Cannon, Alex J. [2,https://orcid.org/0000-0002-8025-3790]; Lima, Aranildo R.[2,https://orcid.org/0000-0003-2514-891X]; Arora, Vivek K.[2,https://orcid.org/0000-0002-2620-9342]; Boulanger, Yan[4]; Melton, Joe R.[2,https://orcid.org/0000-0002-9414-064X]; Van Vliet, Laura[5,https://orcid.org/0009-0003-9868-3486]; Zhang, Xuebin[6]

1: Climate Research Division, Environment and Climate Change Canada, Toronto ON
2: Climate Research Division, Environment and Climate Change Canada, Victoria BC
3: Northern Forestry Centre, Canadian Forest Service, Natural Resources Canada, Edmonton AB
4: Laurentian Forestry Centre, Canadian Forest Service, Natural Resources Canada, Québec QC
5: Canadian Centre for Climate Services, Environment and Climate Change Canada, Victoria BC
6: Pacific Climate Impacts Consortium, University of Victoria, Victoria BC

Supplementary Table 1. Models used in the analysis.

**Ensemble**	**Forcing**		**Model**	**Number of realizations**
CanLEAD-FWI	ALL	historical+RCP8.5	CanRCM4, bias corrected	50
CMIP6-historical	ALL	historical+SSP2.45	ACCESS-CM2	10
CMIP6-historical	ALL	historical+SSP2.45	ACCESS-ESM1-5	39
CMIP6-historical	ALL	historical+SSP2.45	CanESM5	50
CMIP6-historical	ALL	historical+SSP2.45	EC-Earth3	1
CMIP6-historical	ALL	historical+SSP2.45	GFDL-ESM4	1
CMIP6-historical	ALL	historical+SSP2.45	HadGEM3-GC31-LL	5
CMIP6-historical	ALL	historical+SSP2.45	INM-CM4-8	1
CMIP6-historical	ALL	historical+SSP2.45	IPSL-CM6A-LR	11
CMIP6-historical	ALL	historical+SSP2.45	MIROC6	50
CMIP6-historical	ALL	historical+SSP2.45	MPI-ESM1-2-LR	2
CMIP6-historical	ALL	historical+SSP2.45	NorESM2-MM	2
CMIP6-historical	ALL	historical+SSP2.45	TaiESM1	1
CMIP6-DAMIP	NAT	historical-natural	ACCESS-CM2	3
CMIP6-DAMIP	NAT	historical-natural	ACCESS-ESM1-5	3
CMIP6-DAMIP	NAT	historical-natural	CanESM5	50
CMIP6-DAMIP	NAT	historical-natural	HadGEM3-GC31-LL	10
CMIP6-DAMIP	NAT	historical-natural	IPSL-CM6A-LR	6
CMIP6-DAMIP	NAT	historical-natural	MIROC6	50
CMIP6-HighResMIP	ALL	highresSST-present+highresSST-future	CNRM-CM6-1-HR	10
CMIP6-HighResMIP	ALL	highresSST-present+highresSST-future	EC-Earth3P	2

CMIP6-HighResMIP	ALL	highresSST-present+highresSST-future	EC-Earth3P-HR	2
CMIP6-HighResMIP	ALL	highresSST-present+highresSST-future	HadGEM3-GC31-HM	3
CMIP6-HighResMIP	ALL	highresSST-present+highresSST-future	HadGEM3-GC31-LM	5
CMIP6-HighResMIP	ALL	highresSST-present+highresSST-future	MRI-AGCM3-2-H	1
CMIP6-HighResMIP	ALL	highresSST-present+highresSST-future	MRI-AGCM3-2-S	1

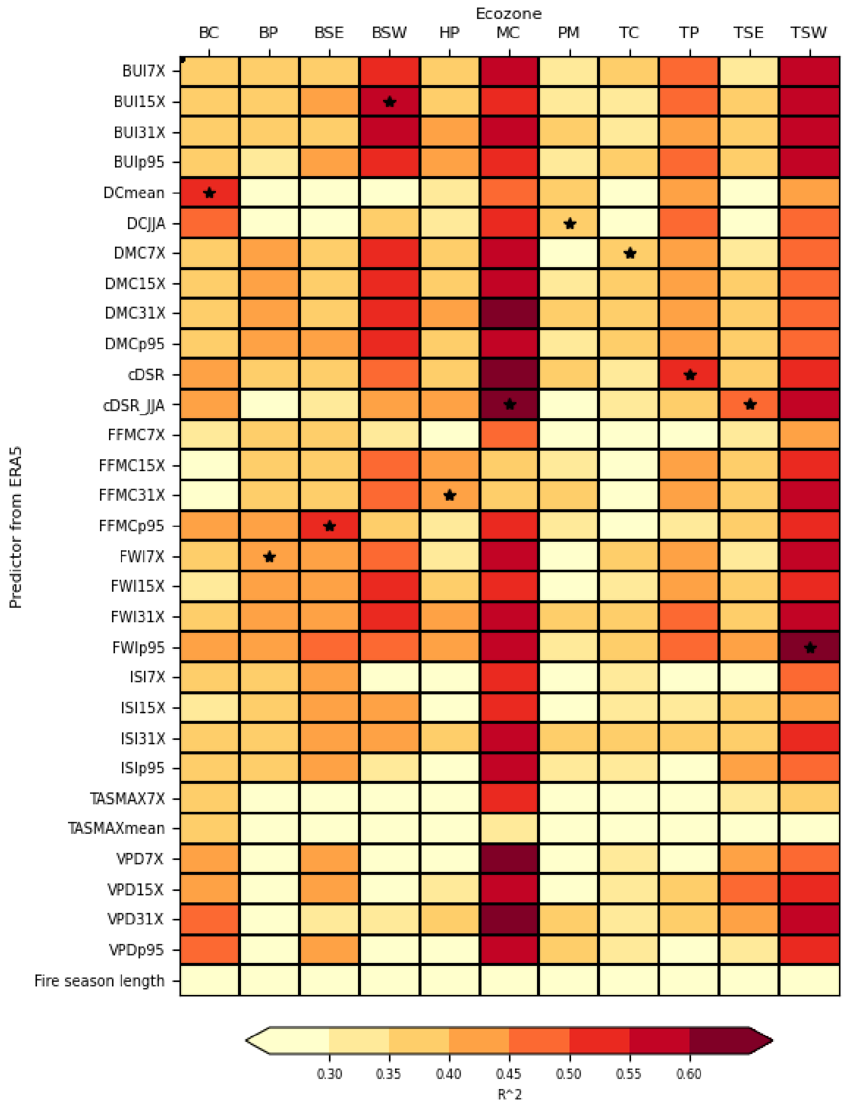

Supplementary Figure 1. Similar to Figure 2a but including all predictors considered. See Figure 2a and accompanying caption for more details regarding the predictor definitions.

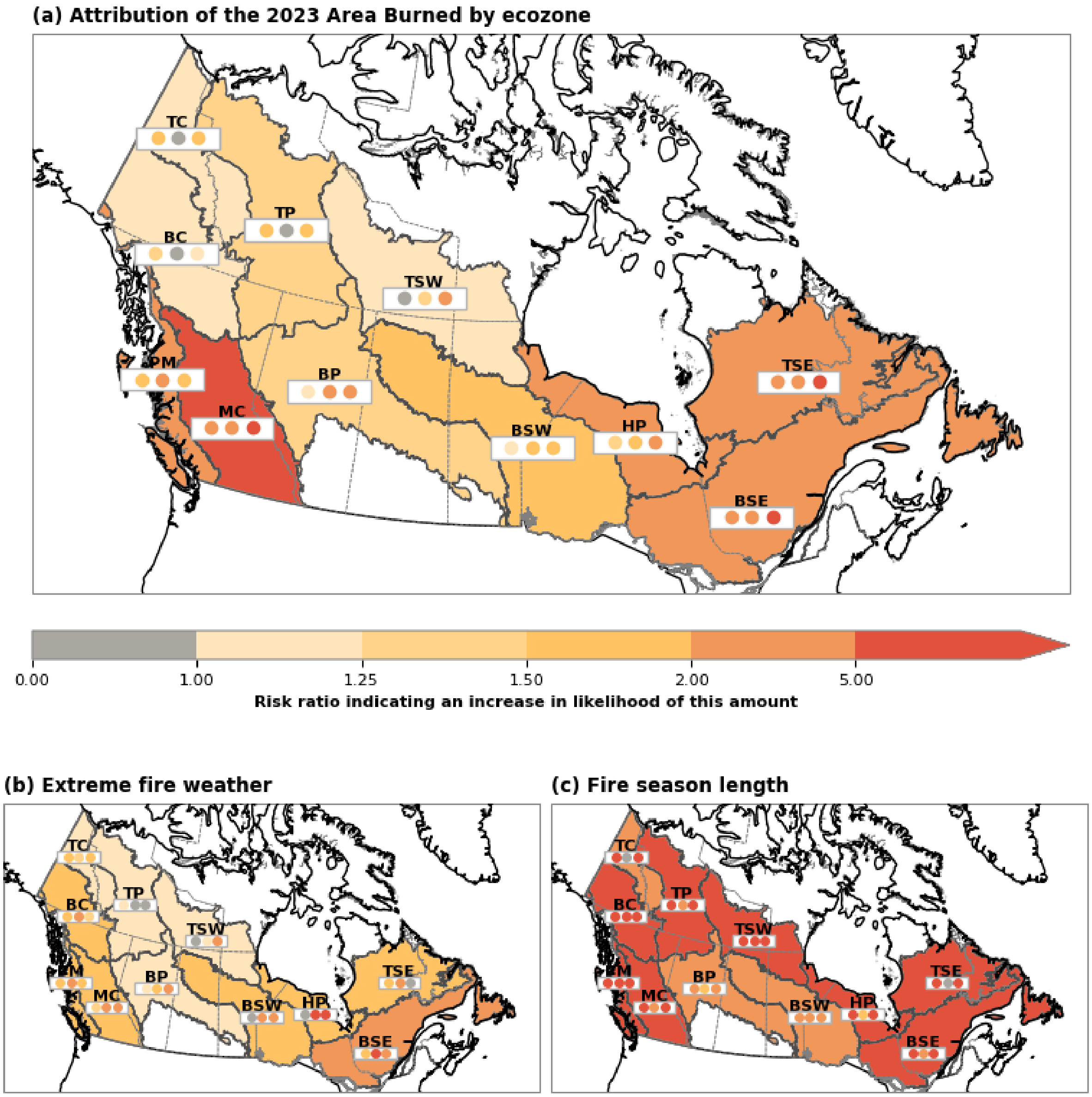

Supplementary Figure 2. Similar to Figure 3 but using the best estimate of the risk ratio.

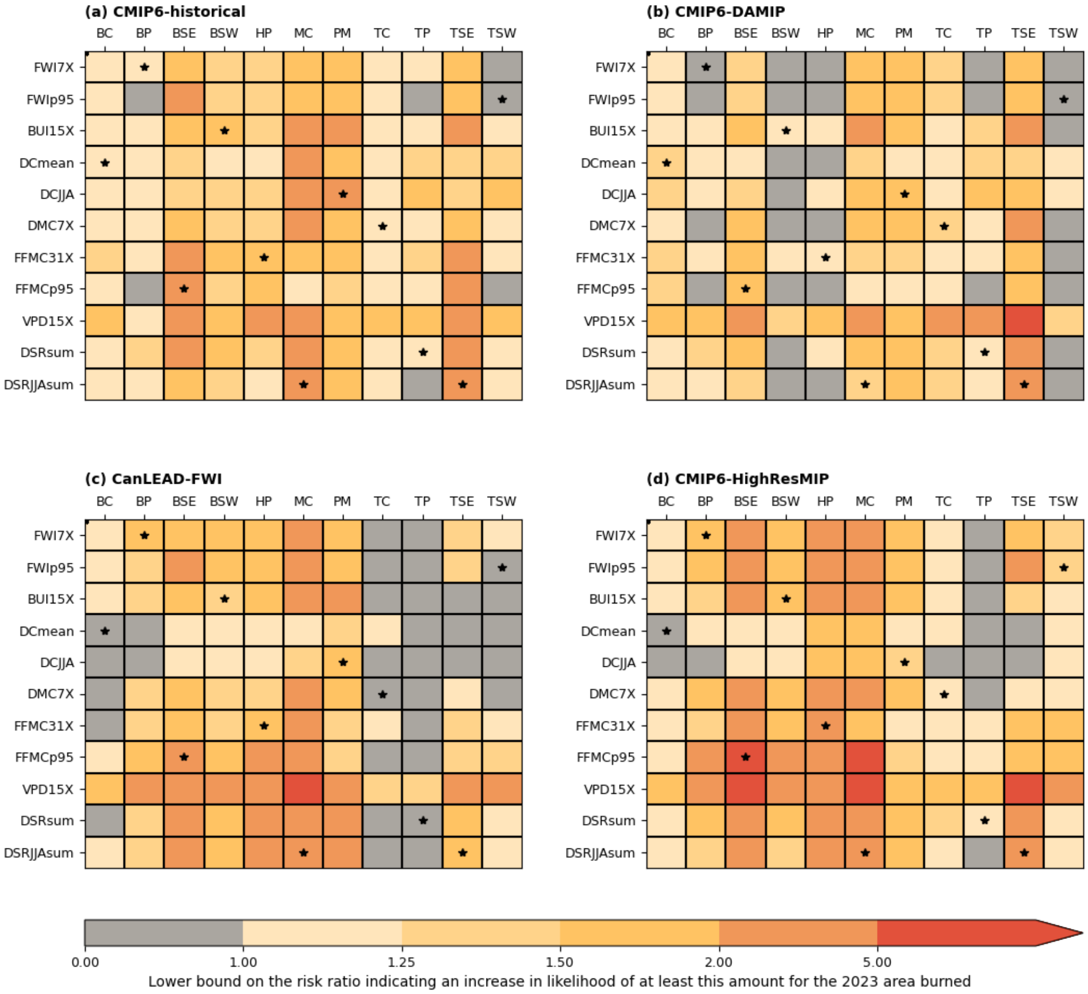

Supplementary Figure 3. Attribution results for area burned by predictor used for the regression. In each panel, columns indicate regions and rows regression predictors. Starred spaces indicate the best predictor that is used for the results in Figure 3. Results are shown for (a) CMIP6-historical (main shading in Figure 3), (b) CMIP6-DAMIP (left circle in Figure 3), (c) CanLEAD-FWI =(center circle in Figure 3), (d) CMIP6-HighResMIP (right circle in Figure 3). Note that (a) and (b) use different sets of model simulations as not all models provided NAT realizations. See Figure 2 for explanations of the predictors.

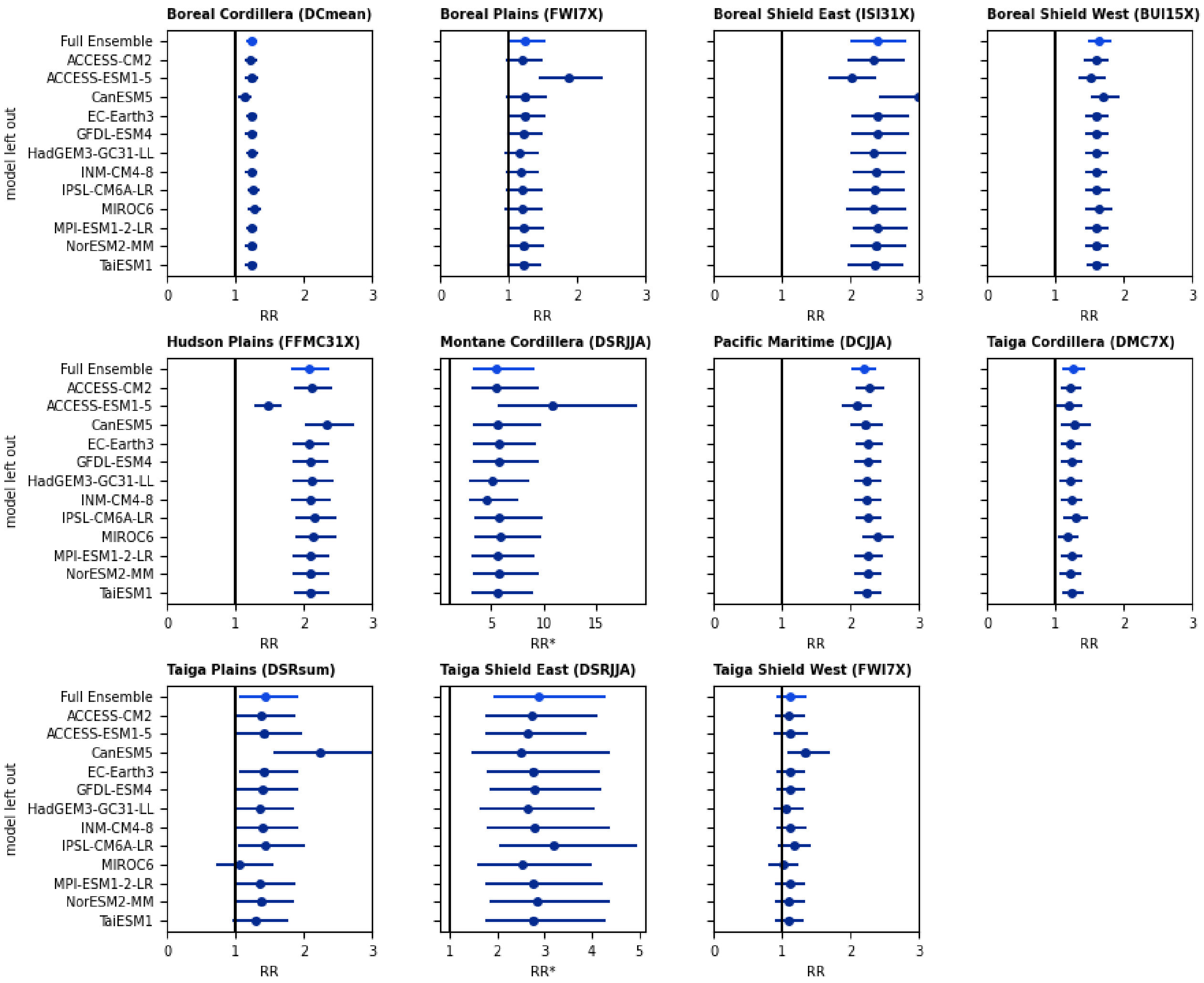

Supplementary Figure 4. Sensitivity of the risk ratio (RR) for area burned and its uncertainty range to individual models. The RR for area burned in each ecozone was calculated using the CMIP6-historical ensemble with all realizations from one model removed. The full ensemble value for comparison is shown in bright blue. If the RR is largely different when one model is removed, this implies that model has a stronger influence, which may be the result of biases. Two regions (indicated with a *) use a horizontal axis scale that allows for larger values. In parentheses following each region name is the best predictor.

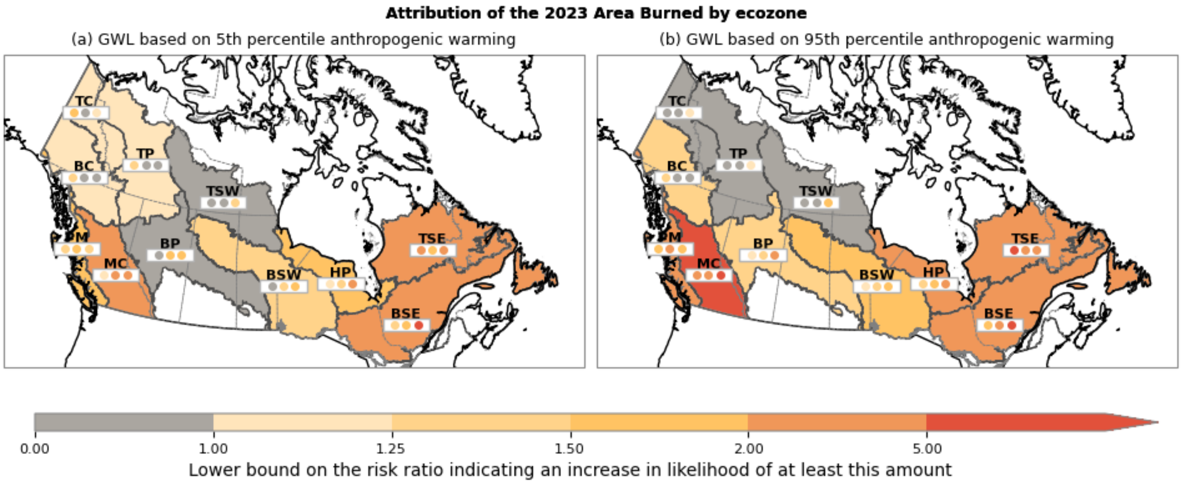

Supplementary Figure 5: Sensitivity of the area burned attribution results to the estimate of human-attributed warming (see Methods). Due to different timing of the warming levels in different models, the sample size for the 5th percentile is larger than the sample size for the 95th percentile.

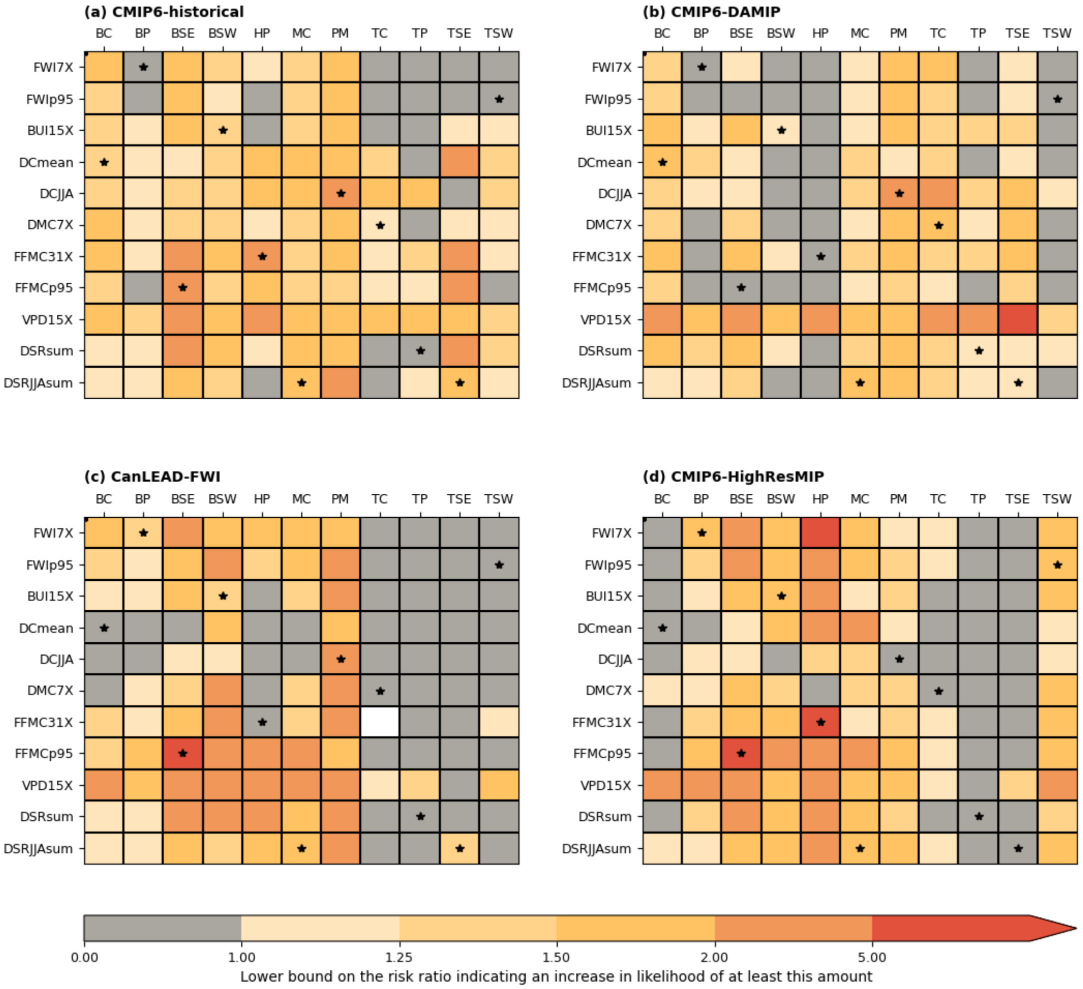

Supplementary Figure 6. Similar to Supplementary Figure 2, but risk ratios are for the indices themselves. Starred indices are the best predictor of area burned in each ecozone. See Figure 2 for the long names and descriptions of the FWI System indices.

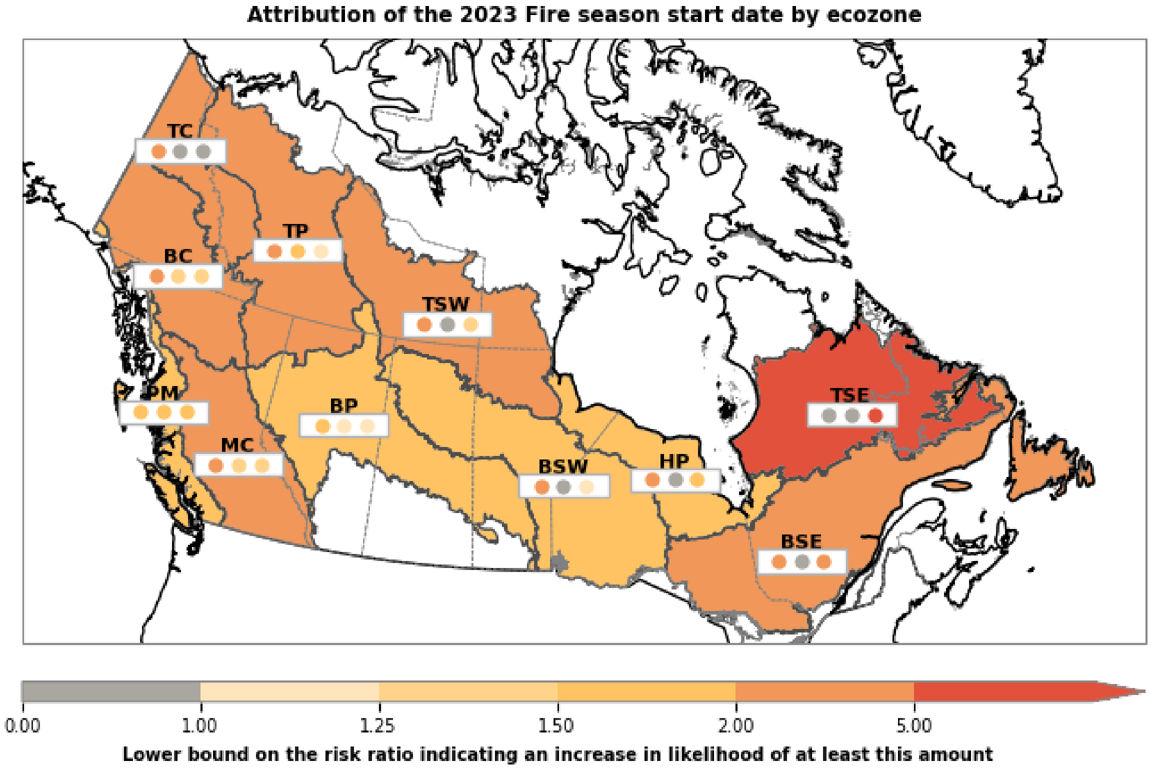

Supplementary Figure 7. As in Figure 3 but for the start of the fire season. Probabilities are for a day of the year equal to or earlier than that observed in 2023.

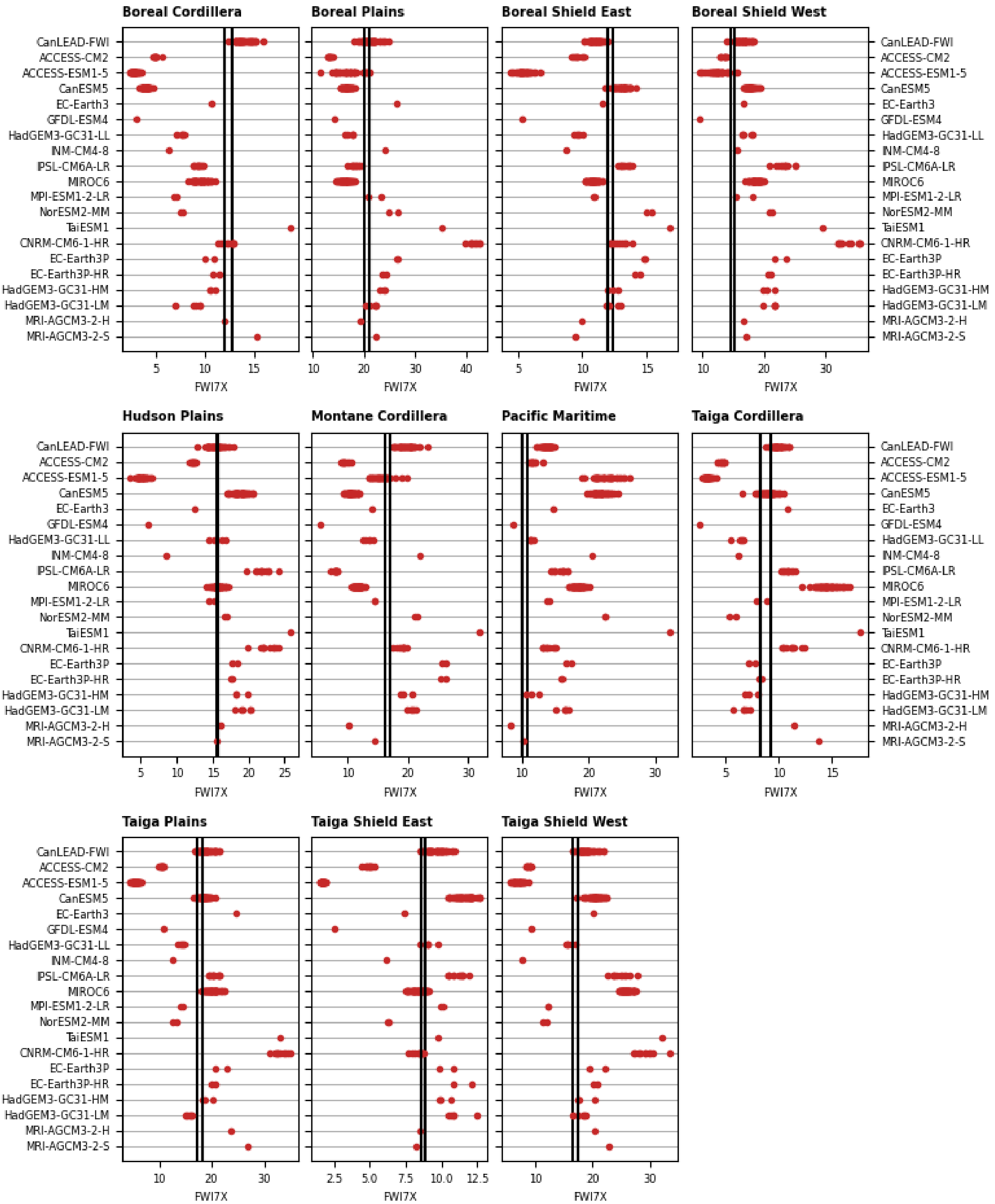

Supplementary Figure 8. By ecozone, the mean FWI7X in the base period 1951-1980 is plotted for each individual model realization. Models included in all three ensembles are shown: CanLEAD-FWI is a single-model ensemble, ACCESS-CM2 through TaiESM1 belong to the CMIP6-historical ensemble and the remaining models are part of CMIP6-HighResMIP. The two vertical bars represent the value from ERA5 for the two different interpolated resolutions (see Methods). A few outlier models were already excluded from the analysis.
